# Exploring the factors influencing public intention for spectator sports consumption based on grounded theory

**DOI:** 10.1038/s41598-024-59049-9

**Published:** 2024-04-08

**Authors:** Fenghao Wang, Junhua Zhou, Chenyu Fan

**Affiliations:** 1 School of Physical Education, Ludong University, Yantai, China; 2 School of Foreign Languages, Ludong University, Yantai, China

**Keywords:** Spectator sports consumption, Grounded theory, Public participate intention, Health care, Risk factors

## Abstract

Spectator sports consumption serves as a vital component in the development of the sports industry. However, numerous challenges exist in fostering public engagement in this domain. Therefore, in order to explore the factors that influence public participation in spectator sport consumption, this study analyzes the intention to participate in spectator sports consumption from the perspective of consumers. On this basis, Semi-structured interviews were conducted with a sample of 25 members of the public, and three levels of coding were analyzed using the qualitative research method of procedural rooting theory and establish a model on the influence of public intention to participate in spectator sports consumption, and on this basis, we reveal the influence of crucial elements. The results of the study indicate that: Firstly, personal and psychological factors are significant internal drivers, while external drivers cover product and contextual factors. Secondly, the key to filling the attitudinal and behavioral gaps is the depth of perception individually, which is of great importance in increasing public participation. Thirdly, external contextual factors impacting consumer support primarily consist of external incentives, social influences, and urban contextual variables, which also serve a moderating role in the integration model. The results suggest that guiding the public to actively participate in spectator sport consumption should be based on an understanding of individual perceptions, emotions as well as attitudes. This paper develops a model examining public motivation to engage in spectator sports locally in China, pinpoints the primary influencing factors and mechanisms, and presents novel concepts for the sustainable growth of the sports sector.

## Introduction

As a burgeoning focal point of China’s economic landscape^1^, Sports consumption has emerged as a vital force propelling the country’s economic expansion^2^. Sports consumption refers to the consumption behavior that people use money to purchase all kinds of sports-related goods and services^3^. Over the past decade, stimulating the vigorous sports consumption and promoting the healthy development of the sports industry has been one of the priorities for the Chinese government. On October 20, 2014, the General Office of the State Council of China issued the “Opinions on Accelerating the Development of the Sports Industry and Promoting Sports Consumption”, which put forward development goals for the sports industry and highlighted the role of sports consumption. On September 17, 2019, the State Council released the “Opinions on Promoting National Fitness, Sports Consumption and the High-Quality Development of the Sports Industry” to strengthen support for the sports industry, stimulate market vitality and enthusiasm for sports consumption. It can be seen that sports consumption is an significant force in driving domestic demand and ensuring China’s economic growth. Boosting sports consumption also serves as a critical approach to enhance public welfare and construct a healthy China^4^.

As a fundamental driver of economic development, sports consumption encompasses three distinct categories:spectator sports consumption, participatory sports consumption, and physical sports consumption. Physical sports consumption involves the acquisition of tangible sports goods, such as sports equipment and apparel. Participatory sports consumption also includes the purchase of sports-related services, such as health counseling, gym memberships, and participation fees for sports events^3^.Spectator sports consumption is the purchase of admission tickets, participation qualification, etc. for the purpose of watching and enjoying various consumer behaviors^5^.The consumption of spectator sports is essential for fostering an uptick in sports consumption and the high-quality growth of the sports business, according to the experience of industrialized nations in Europe and the United States^3^.

Data shows that from 2014 to 2020, China’s spectator sports consumption as a percentage of overall sports consumption among adults and the elderly rose from 5.2 to 7.7%. However, the scale of spectator sports consumption is not only lower than that of developed sports countries such as those in Europe and the United States, but also lags behind China’s own physical and participatory sports consumption^5^.Specifically, this is manifested in the following ways: First, the total consumption is small. On the one hand, it falls below the standard of industrialized nations in Europe and the US. For example, in 2017, the total spectator sports consumption in the United States amounted to 56 billion U.S. dollars, accounting for 56% of sports consumption^6^. In comparison, in 2020, spectator sports consumption by adults and the elderly in China accounted for only 7.7% of total sports consumption^5^; On the other hand, spectator sports consumption is lower than national physical and participatory sports consumption in China. In 2020, physical and participatory sports consumption accounted for 53.7% of total sports consumption for adults and 20.6% for seniors. In comparison, spectator sports consumption only accounted for 7.7% of the total^5^.Second, China’s per capital expenditure on spectator sports is low compared to other countries. For example, per capital sports consumption in the U.S. and Europe generally exceeds $400. In 2017, per capital sports consumption in the U.S. was about $224. In contrast, in 2019 the total per capital sports consumption in Jiangsu, China was ¥2,442.37, with only ¥111.37 spent on tickets and ¥47.49 on broadcasts and recordings of matches^7^.

It is of vital importance to raise public awareness of spectator sport consumption. Jia^3^ used documentation method to study the Development Process and Current issues of Spectator Sports Consumption in China; he suggested that to develop spectator sports consumption further, China needs to: Increase consumption levels; Raise consumer awareness; Improve the quality of sports events and broadcasts and Ensure a safe viewing environment. In addition, Huang^8^ study how Artificial Intelligence developments impact spectator sports consumption. The advancement of spectator sport engagement, as a type of consumption, requires factoring in public intention and backing. However, research on this subject remains in preliminary stages.

Based on the aforementioned analysis, the present research adopted a grounded-theory approach to further identify the factors influencing consumer intention about spectator sports consumption with open coding, axial coding, and selective coding. It chose Yantai city in Shandong province as a typical case to carry out this research, thus, to construct a motivation model about public intention. Furthermore, such study could enrich existing research about spectator sports consumption from a new perspective. Meanwhile, it can lead the government and other stakeholders to vigorously develop the consumption of spectator sports and further catalyze the advancement of the nation’s economic landscape.

The marginal contribution aims at constructing a motivation model on public intention for spectator sports consumption, and exploring the major influencing factors and mechanisms. Firstly, in the light of the interview data, an integration model from individual, psychological, contextual and product dimensions was established. In addition, the logical relationships among the factors, providing a sound theoretical framework was also revealed. Additionally, this study enhanced the mechanism of key elements associated with consumers’ intention, underscoring the directive function of psychological, contextual, and product factors.

The organization of this paper is delineated as follows: The first part is to introduce the background of this study, the second part reviews the literature related to the spectator sports consumption. Subsequently, the methodology and methods adopted for this research are presented in the third part. The motivation model is explained in the forth section and the conclusion in the fifth section.

## Literature review

### Sports consumption

The scholarly inquiry into spectator sports consumption traces back to the waning years of the twentieth century. People are beginning to realize that sport is not only a culture but also a form of consumption^9^. Early theoretical and empirical studies have contributed to the deepening of scholars’ perceptions of the relationship between consumption and sport^10–13^, but most of these studies have been dedicated to the influencing of the consumption to a particular item on sport, neglecting the consumption of sport itself. Since then, scholars have begun to explore the impact of sports consumption on the sports industry and the structure and status of sports consumption in different regions and groups from sports consumption itself^14–17^. Theoretical research on sports consumption lays the groundwork for developing spectator sports in China. As China’s economy grows rapidly, the sports industry is evolving its development models and environments accordingly. Sports consumption, a major component of the sports industry, presents new opportunities to advance China’s sports sector and broader economy^18^. Currently, the scholarly examination of sports consumption in domestic and international literature principally concentrates on the subsequent facets:

Firstly, the prevailing literature concentrates on the characteristics of residents’ sports consumption in different regions from a geographical perspective, summarizing the habits and characteristics of residents’ sports consumption in various regions. Wang Y et al. used the Double-hurdle model to analyze the influencing mechanisms about Beijing residents at two decision-making stages: participation in sport consumption and sport consumption expenditure^19^. Li G et al. used the Expanded Linear Expenditure System model (ELES model) as an analytical tool to analyze the characteristics and development trends of the sports consumption structure of rural residents in the Yangtze River Delta region^20^. Secondly, the prevailing literature mainly be intent upon different sports consumers.Fridley A et al. examined differences in the motivations of marginalized college students to consume sports and found that the dominant group scored significantly higher than the combined marginal group on four of the eight sports consumption motivations studied^21^. Wang X et al. used the ELES model to estimate the parameters and analyze the marginal propensity to consume and elasticity of demand of the two-stage sports consumption structure of landless peasants in Changsha in 2012^22^. Zheng H et al. take permanent residents of Shanghai who are 18 years old or older as the research object, and propose the structure of the constraint and promotion system that affects residents’ demand for participatory sports consumption from the perspective of system theory^23^. Thirdly, existing studies examine hotspots of sport consumption in different development contexts. Drawing upon the fundamental theoretical framework of industrial economics, Ren B et al. explores the logic, motivation and path of high-quality development of China’s sports industry under the new development pattern of the “double cycle”^24^. Chan-Olmuted S et al. investigates the impact of participation in fantasy sports on sports media consumption in the context of rapidly evolving new media and consumer viewing habits^25^. Liu J et al. use literature, logical analysis and other methods to study the connotation, development characteristics and dilemmas of the new consumption of sports in the digital era, and put forward the practical way forward^26^. Fourthly, existing literature on the development of sports consumption problems in the process of research, for different problems scholars have given their respective insights and countermeasures. Thibaut E et al. used a two-step Heckman approach to explore the determinants of household sport consumption^27^. Yan et al. explored the constraints of sports consumption upgrading in China under the new economy and the path of realization^28^. Using literature and other research methods, Liu G analyzes the literature on physical activity, sports consumption and the impact of population aging among the elderly in the past 20 years, and suggests that the study of relevant basic concepts should be strengthened and the quantitative study of the impact of population aging on sports consumption should be reinforced in future research^29^.

### Sport consumption intention

Consumption intention is an antecedent variable for conducting consumption behavior and plays a key role in influencing consumers to make purchase decisions. According to Chen SP, sports consumption willingness refers to an individual’s comprehensive intention judgment on whether sports consumption is valuable or not and whether he or she is willing to pay a monetary price to participate in sports behavior^30^. Liu Y et al. believe that sports consumption willingness reflects the subjective tendency of consumers to choose a specific consumption, which is a pre-variable of consumer behavior^31^. Generally speaking, the stronger the intention to consume sports, the more sports consumption behaviors consumers will participate in. Currently, the scholarly investigation into sport consumption intention in domestic and international literature chiefly examines the subsequent facets:

Firstly, the existing literature has studied the influencing factors of sports consumption intention. Zhang GM points out that people’s preferences are different, the choice of sports products and services is therefore different, and people’s preferences are influenced by factors such as the economic environment, social environment and cultural environment^32^. Zhang XP believes that the factors affecting the demand for sports consumption are influenced by government policy factors in addition to consumer income, consumer preferences, sports commodity prices and related commodity prices^33^. The National Fitness Program and the national policy of stimulating domestic demand have increased the total demand for sporting goods to a certain extent. Secondly, existing studies have been conducted on the sports consumption intention of specific groups of people. Chen F argues that in China, sports consumption shows a clear class gap, which is larger than the gap in residents’ economic income, and the resulting gap in social and cultural resources clearly has an impact on sports consumption^34^.Zhang James et al. explored the relationship between “push and pull” factors and fans’ consumption of sports, using women’s professional basketball games as an example.^35^. Thirdly, existing studies have examined sports consumption intention based on a particular variable. Through an empirical study, Zhu ZY found that self-presentation had a positive effect on sport consumption intention based on content exposure in online social networking^36^.Funk DC et al. explored the relationship between sport consumer motivation and different variables based on self-determination theory. The study findings reveal that sport consumer motivation encompasses both intrinsic and extrinsic motivations. Intrinsic motivation reflects behaviors where individuals engage in sport consumption as an end in itself, while extrinsic motivation involves participating in the activity with the goal of attaining a separable instrumental outcome.^37^.

### Spectator sports consumption

One of the types of sports consumption is spectator sports consumption^3^. Spectator sports consumption refers to the purchase of admission tickets, participation qualification, etc. for the purpose of watching and enjoying various consumer behaviors^5^. Currently, the scholarly investigation into spectator sports consumption in domestic and international literature chiefly examines the subsequent facets:

Firstly, the prevailing literature concentrates on the impact of different factors on the consumption of spectator sports. Huang J explores how the development of artificial intelligence for spectator sports consumption based on Internet usage data^38^. Ma P et al. investigated the influence of family and social factors on residents’ live spectator sports consumption under the perspective of consumption embeddedness^39^. In order to investigate the internal driving mechanism influencing Chinese consumers’ loyalty to spectator sports, Xun Y et al. constructed and tested a mechanism model of the influence of loyalty to spectator sports through structural equation modeling with product involvement as the key variable^40^. Using NCCA women’s college basketball as an example, Trail GT et al. explored the factors that influence the consumption of spectator sports^41^. Yi XR et al. explored the influence of personal values on participation in spectator sport consumption^42^. Secondly, the prevailing literature concentrates on the development of spectator sport consumption from a single perspective. Jia W analyzes the development history, practical problems and development strategies of spectator sports consumption in China by using the literature method and other methods^3^. Zhang J explores the sustainable development of China’s spectator sports consumer market in the context of a moderately affluent society^43^. Thirdly, existing research has explored the development of a consumer market for spectator sports. Kang J et al. explored the factors constraining the development of the spectator sports consumer market in China^44^. Liu W et al. explored the supply and demand for the development of the spectator sports consumer market^45^. Liu W et al. analyzed the supply and demand characteristics of China’s spectator sports consumer market through their research^46^. Fourthly, existing literature has explored the relationship between spectators and sports media consumption. Koronios K et al. used a quantitative research approach to explore the effects of motivation and constraints on fans’ participation in media sports consumption^47^. Koronios K et al. analyzed the link between motivation, constraints, and sports media consumption using STATA^48^. Chiu WS et al. used structural equation model to explore the relationship between SFE, identification (with players and teams) and media consumption intentions^49^.

**Figure 1:**
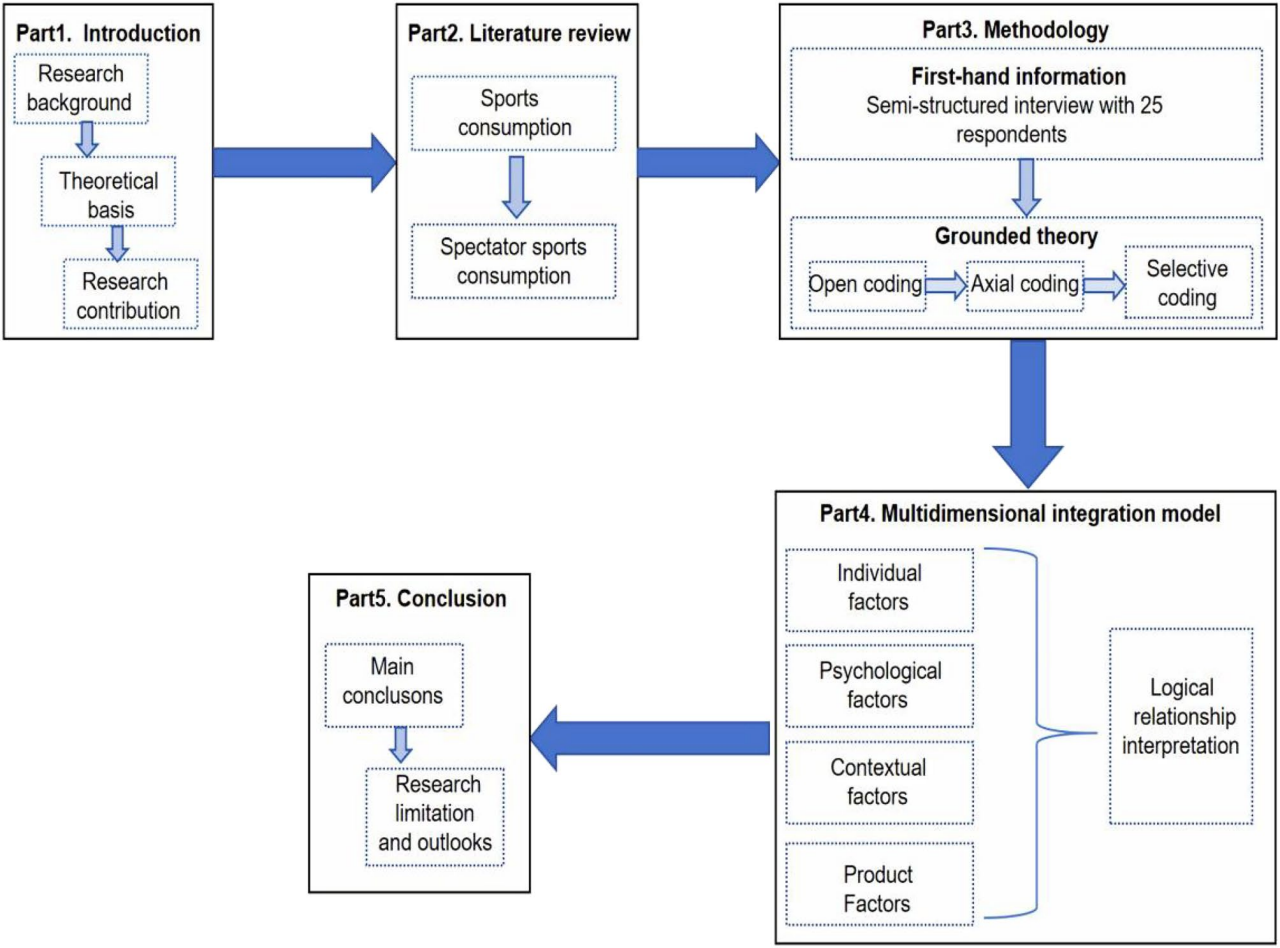
The research framework.

As a consumption pattern, the public’s intention to participate in spectator sports consumption is directly related to its participation or non-participation. At present, the research on spectator sports consumption mainly focuses on the development strategy of spectator sports consumption, influencing factors and the development of related consumer markets, but lacks the research on the public’s intention to participate in spectator sports consumption. Although a small amount of literature has explored the connection between spectators and sports consumption, it is only conducted from the perspective of the media’s consumption of sports, the attendance rate of the games, and other specific aspects and special groups such as fans of a certain sport. Perspective, without going deep into the grassroots to understand the general public’s intention to consume sports. Therefore, based on the research of scholars at home and abroad, this study utilizes the research method of rooting theory, adopts three-level coding, and goes deep into the grassroots to identify the factors that affect the public’s intention to participate in spectator sports consumption. Rooted theory is a qualitative research method, which is usually applied by researchers to establish theories, and researchers often do not study theories before they start, so their findings are more innovative. The research framework is shown in Fig. 1.

## Methods, material, and analysis

### Research methods

The lack of research on spectator sports consumption has resulted in a deficit of theory elucidating consumers’ intention. On this matter, case studies is able to offer abundant practical evidence to address questions in the aspect of “how” and “why”, and to demonstrate the interconnections between ideas and reveal logical relationships^50^. There’s no doubt that grounded theory is an empirical methodology that facilitates both adaptable qualitative research and theoretical investigation, thereby enhancing reliability and validity^51^. Accordingly, utilizing a multi-case approach based on grounded theory, this study investigated public’s intention for spectator sports consumption and sorted out feasible approaches for improving such intention.

After a long period of development, three major schools of thought on grounded theory have now emerged: classical, procedural, and constructivist^52^. Different theoretical schools of thought have reached a basic consensus in terms of iterating through the information, but there are significant differences in the coding sessions. Classical grounded theory is divided into two main steps: substantive coding and theoretical coding; Procedural grounded theory includes open coding, spindle coding, and selective coding; Constructivist grounded theory, on the other hand, consists of four steps: initial coding, focused coding, axial coding, and theoretical coding. Performing spectator sport consumption can be understood as a process of constructing the self, reality and society through multiple human interactions with technology and sport^53^. Programmed grounded theory, with its philosophical background in pragmatism and symbolic interaction theory, emphasizes the role of the researcher in interpreting the behavior and the meaning of human interaction. Through the programmed rooting theory, researchers can explore relationship between concepts, discover the influence mechanisms behind the problems, and scientifically explain the occurrence mechanism of the public’s intention of spectator sports consumption.

Therefore, based on the school of procedural rooting theory, this study adopts the research paradigm of “open coding-major axis coding-selective coding” to comprehensively and systematically explore the influence model of the public’s intention of spectator sports consumption, in order to enrich the research methodology and theoretical foundation of this field.

All methods were carried out in accordance with relevant guidelines and regulations.

### Ethics approval

The interviewees designed for this study were approved and supported by the School of Physical Education and Sport at Ludong University. Subjects participated voluntarily and provided written informed consent.

### Consent to participate

Informed consent was obtained from all individual participants included in the study.

### Material

The material in this paper is drawn primarily by semi-structured interviews which are designed to elicit information that affects the public’s intention of spectator sports consumption. The semi-structured interview framework was developed by reviewing existing literature and incorporating suggestions from some experts and scholars. Furthermore, we randomly selected three interviewees to conduct a pre-survey, based on whose feedback we modified the preliminary interview framework. This process led to the finalization of the formal interview framework^54^.

The sampling of rooted theory is theoretical sampling which requires the researcher to determine the next step in how to draw the research subjects and collect data on the the basis of the results on the current analysis^53^. In the initial interviews, a wide range of respondents of different ages, genders, occupations and regions were selected for open-ended interviews, e.g. Are you aware of spectator sport consumption? Do you participate in spectator sport consumption? Subsequently cascading, for interviewees who participant in spectator sport consumption, we further asked them questions about the type, scope, as well as the frequency of their participation. Meanwhile, for non-participants, we explored the influencing factors that affect their intention to participate. After a number of comparisons and collations, the analysis of the data no longer resulted in new category relationships and the work of expanding the categories of the interviews was discontinued.

In order to ensure the cooperation of the respondents and the reliability and representativeness of the data, this study requires the respondents to have a certain understanding and knowledge of the issues under study, and we chose the respondents who are in the age bracket between 18–55 years old, come from different occupations and have a regular income from the public. The sample size was determined according to the guidelines of theoretical saturation. Finally, a total of 25 respondents were selected for this study. Interviews were conducted with each respondent either face-to-face or by video interview. Each interview will last for 20–30 min. The whole interviews were audio-recorded and the recordings were converted to text at the end of the interviews. The basic profile and information of the interviews is presented in Table 1**.**

**Table 1:** Basic Information of the Subject.

No	Gender	Age	Territory	Qualification	Address	Careers	Monthly salary
A1	Female	19	City	College	Shandong	Student	1500￥
A2	Female	33	City	High school	Hebei	Musician	8000￥
A3	Male	20	Village	College	Shandong	Self-publisher	6500￥
A4	Male	38	City	High school	Hebei	Construction industry personnel	11,000￥
A5	Female	27	City	Junior college	Xinjiang	Freelance	7500￥
A6	Male	24	Village	College	Beijing	Internet worker	10,000￥
A7	Female	29	Village	Junior college	Hebei	Company employee	8000￥
A8	Male	18	City	College	Shandong	Student	1300￥
A9	Male	26	Village	Junior college	Xinjiang	Company employee	6500￥
A10	Male	36	City	High school	Hebei	Company employees	9200￥
A11	Female	29	Village	Postgraduate	Beijing	Research worker	8000￥
A12	Female	42	Village	Middle school	Shandong	Agricultural occupation	7500￥
A13	Male	23	City	Junior college	Shandong	Company employee	5800￥
A14	Male	31	City	Postgraduate	Hebei	College professor	13,000￥
A15	Female	38	City	High school	Beijing	Fitness instructor	8000￥
A16	Male	29	Village	Junior college	Harbin	Professional institution	7800￥
A17	Male	32	City	College	Shandong	Secondary school PE teacher	9500￥
A18	Female	29	Village	Junior college	Shandong	Nurses	5300￥
A19	Male	55	City	Middle school	Beijing	Service Industry	14,000￥
A20	Male	49	Village	Middle school	Hebei	School logistics	7400￥
A21	Male	34	Village	College	Beijing	Research worker	10,000￥
A22	Female	46	City	High school	Hebei	Psychological Counselor	6000￥
A23	Female	36	Village	College	Harbin	Suppliers of sports equipment	35,000￥
A24	Male	47	City	Middle school	Beijing	Logistics worker	18,000￥
A25	Male	21	Village	Junior college	Hebei	An investment banker	12,000￥

### Data analysis

#### Open coding

Open coding is primarily used to discover categories and concepts of the research question. To minimize bias, open coding requires the researcher to review, compare, and interpret the source material verbatim without any theoretical presuppositions or subjective without any theoretical preconceptions or subjective bias, and to review, compare, and organize them^53^. Following this principle, this study first summarized the key information from 25 interview transcripts by removing irrelevant content, yielding a total of 324 valid original statements. Next, the original statements were abstracted into concepts, while eliminating initial concepts that appeared only once or were contradictory. In the end, 46 initial concepts were obtained, each supported by corresponding original statements.

In order to connect the concepts formed by open coding, this paper further organized and summarized the connotations between the initial concepts, and finally extracts 20 subcategories. On this basis, the 20 subcategories were further abstracted to obtain eight main categories. Based on this, the 20 subcategories were further abstracted and 8 main categories are obtained. In summary, 46 initial concepts, 20 subcategories and 8 main categories were obtained through open coding. The open coding results correspondingly were presented in Table 2**.**

**Table 2:** Open Coding Results.

Main category	Father node	Child node
A1 Subjective cognition	B1 Sports cognition	b1 Sports awareness
		b2 Sports behaviors
	B2 Values	b3 Social-interested values
		b4 Self-interested values
A2 Consumer attributes	B3 Individual characteristics	b5 Consumer earning
		b6 Consumer demand
		b7 Level of love for sports
	B4 Family characteristics	b8 Family earning
		b9 Family structure
A3 Social influence	B5 Sports environment	b10 natural conditions
		b11 Number of sports events
	B6 Social capital	b12 Profit
		b13 Social relationship
		b14 Social networks
	B7 Peer influence	b15 Interactive exchanges
A4 Consumer perception	B8 Feeling	b16 Relaxation
		b17 Comfortable
	B9 Consumption cognition	b18 Consumption backgrounds
		b19 Consumption goals
		b20 Consumption results
		b21 Viewing methods
A5 Behavioral motivation	B10 Emotion motivation	b22 leisure and entertainment
		b23 socialization
		b24 self-fulfillment
	B11 Learning motivation	b25 Access to information
		b26 Get Sports Information
		b27 Learning the techniques
A6 External motivation	B12 Policy care	b28 Policy-oriented
		b29 Venue design
		b30 Policy implementation
		b31 Subsidy policy
	B13 Marketing incentives	b32 Publicity efforts
		b33 Celebrity effect
		b34 Infusion of technology
	B14 Information content	b35 Too much information
		b36 Low quality of information
A7 Product attributes	B15 Event attributes	b37 Safety performance
		b38 Event level
	B16 Cost	b39 Use cost
		b40 Other cost
	B17 Competition level	b41 degree of enjoyment
A8 Urban contextual	B18 Development level	b42 Economic development
		b43 Input
	B19 Facilities configuration	b44 Infrastructure
		b45 Sports ground
	B20 Natural environment	B46 Geographic conditions

#### Axial coding

The categories generated by open coding are usually independent and dispersed. Therefore, after the initial categories are extracted, it is necessary to further clarify the connections between the categories through principal axis coding, and then mine the higher-level main categories. Accordingly, this paper abstracts and correlates the 20 subcategories and 8 main categories formed by the open coding.

**Figure 2:**
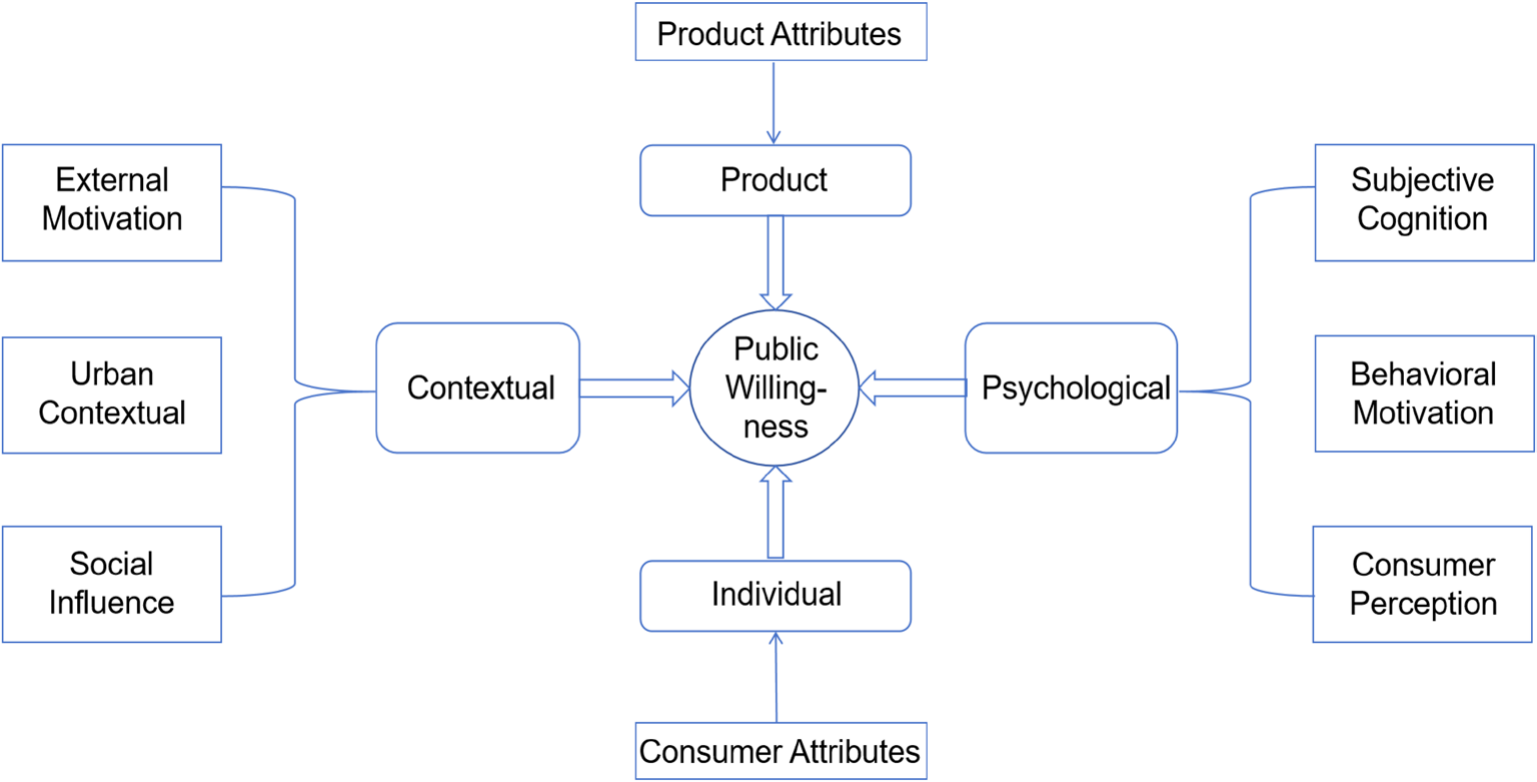
Model of factors about participation intention of public spectator sports consumption.

The individual-psychological-contextual interaction is regarded as the cause of behavior by social psychology^55^. Nevertheless, the object of spectator sport consumption is a specific product, hence the product factor should also be considered as a major dimension. As a result, this study further distills the eight main categories into four factors: individual factors, psychological factors, contextual factors, and product factors. Using grounded theory, we collected and analyzed the data obtained from semi-structured interviews. According to the results, a motivation model was developed to examine the elements influencing public’s intention to consume for spectator sports (See Fig. 2).

#### Selective coding

Selective coding is the development of a systematic theoretical modeling framework by extracting the dominant core categories from the main categories, and discovering typical relationships and “story lines” between the core categories and the main categories, as well as between individual categories. This paper constructed a typical relational structure among the categories of this study based on repeated comparative analyses of the relationships among the existing eight main categories and four core categories (See Table 3).

**Table 3:** The typical relationship structure of the main category.

Typical Relationship Structure	Definition
Individual factors→ public intention	Individual determinants represent the intrinsic aspects that influence consumers’ receptiveness to engage in spectator sports consumption, which affect the public’s intention directly
Psychological factors→ public intention	Psychological factors represent the intrinsic aspects that influence consumers’ receptiveness to engage in spectator sports participation, which affect the public’s intention directly
Contextual factors→ psychological factors public intention	Contextual factors are the external elements that affect consumers’ intention to participate in spectator sports consumption, which has an impact on the magnitude and orientation of the association between psychological determinants and public receptiveness
Product factors→ psychological factors public intention	Product factors are the external elements that affect consumers’ intention to participate in spectator sports consumption, which has an impact on the magnitude and orientation of the association between psychological determinants and public receptiveness

#### Theoretical saturation testing

Grounded theory emphasizes that theory saturation is naturally occurring rather than verified. Therefore, this study analyzes the model structure with a naturally occurring test of theory saturation based on the methodology of Francis et al^56^. Specifically, a total of 25 respondents were interviewed for this paper, and by the time the coding of 22 respondents’ interview data was completed, no new concepts or relationships had emerged, and three additional interviews were subsequently coded to confirm that this was indeed the case. It can therefore be assumed that data coding for this study was saturated.

## Explanation of motivation model for public intention for spectator sports consumption

The foregoing analysis substantiated that the Individual-Psychological-Contextual-Product factor integration framework can sufficiently explain the constitutive process underpinning consumers’ receptiveness to participate in spectator sports consumption. Particularly, the influencing factors can be categorized into four dimensions: individual, psychological, situational and product factors. However, the mechanisms through which these factors influence intention are not uniform. The varying mechanisms of these factors will be analyzed in greater detail below.

### Individual factors

The findings revealed that personal attributes and familial attributes were the consumer characteristics impacting intention for spectator sports consumption. These internal factors were identified as integral in bolstering participation intention, with an inherent causal association apparent between consumer qualities and participation intent.

Variations in individual characteristics—including gender, age, income, hobbies, and other demographic traits—primarily account for differences in reactions to identical stimuli^57^. Sports-focused consumers are more likely to engage in spectator sports consumption. Thus, the intrinsic motivation for increasing intention to participate in spectator sport consumption is to enhance consumers’ sport consciousness. Variations in consumer behavioral characteristics also account for attitudes to spectator sport consumption. Habitual consumer behavior may lead consumers to prefer physical sports consumption and therefore resist spectator sports consumption. However, for impulsive consumers, the strong sports atmosphere of a sporting event is more attractive, and thus, they are more likely to choose spectator sports consumption.

Family characteristics are the significant dimension of consumer characteristic. The impact of family background on the intention to consume spectator sports cannot be ignored. The family life-cycle theory delineates five discrete stages ranging from family formation to bereavement: bachelorhood, newlywed, full-nest, empty-nest, and widowhood. Individuals occupying diverse life-cycle phases have disparate consumption requirements and inclinations, engendering variances in behavioral aims and procuring tendencies^58^. For example, “My parents are both sports practitioners. They have taken me to watch different sports events and taught me many sports. Therefore, spending on spectator sports has become a spending habit for me”.

### Psychological factors

The psychological determinants of consumers have a vital function in catalyzing the evolution of the sports industry. Likewise, the public’s propensity for spectator sport consumption is intrinsically tied to psychological determinants. The findings indicate that subjective cognizance, motivational drivers, and perceptual inclination were the predominant psychological factors influencing consumer intention for spectator sports consumption.

#### Subjective cognition

The theory of planned behavior posits that behavioral intentions are molded by three cardinal predictors: attitudes, personal norms, and perceived behavioral agency^59^. The interview findings revealed that consumers’ subjective cognition is intrinsically linked to their intention for spectator sport engagement. Through open coding, the preliminary categories of subjective factors were classified into values and sporting perceptions.

The respondents exhibited divergent attitudes toward spectator sports consumption based on their distinct values. In particular, sport-minded individuals have a strong desire to participate in spectator sport consumption. They believe that they can obtain psychological relaxation and emotional release from it. Conversely, for individuals espousing pragmatic values, their intention was more susceptible to external influences^54^.

Participation in spectator sports consumption is a consumption behavior that reflects consumers’ perceptions of sports. Therefore, it is critical to accurately assess the impact of sports awareness on the intention to participate in spectator sports consumption. Sports consciousness is the reflection of the objective sports phenomenon in the human brain, which is the synthesis of people’s feelings, thinking and judgment about sports^60^. Sport consumption awareness is an individual’s value-added or not for sport consumption comprehensive judgment and behavioral intention of whether they are willing to spend money in sport activity literature orientation^61^.The behavioral intention model (theory of justified behavior) suggests that behavior is the result of a particular intention^62^.Thus, individuals with high sports awareness tend to be more cognizant of sports consumption and more inclined to participate in spectator sports activities.

#### Behavioral motivation

Motivation constitutes an intrinsic impetus that propels individuals toward the actualization of their objectives^63^. Consumer conduct is contingent on the interplay between diverse motivational impetuses and deliberative determinants, and there is an association between different motives and behavioral outcomes^64,65^. The main intrinsic drivers for participation in spectator sport consumption include emotional motivation and learning motivation.

Comprehending consumers’ proclivity for spectator sports consumption is a multifaceted affair, wherein emotional impetus constitutes one determinant in the intricate web of influencing variables. Driven by emotional motivation, consumers will behave accordingly in order to satisfy their needs and achieve their goals. For example, “I think that by watching sporting events you can make a lot of friends who share a common interest.” “My father and grandfather both supported the team, so I have carried on the family tradition of continuing to support the team and going to the games to cheer them on.”

After one’s physiological needs are satisfied, there is a need for further learning and self-actualization. Learning motivation motivates consumers to buy and learn from quality products and knowledge recognized by others and themselves, so as to acquire the knowledge they need.For example, soccer coaches watch high-level soccer matches to learn the offensive and defensive tactics of soccer, as well as the technical movements of players at different positions. Low-end tournament operators can improve the quality of their tournament organization and operation by watching high-level sports events on site to learn the details of tournament management such as tournament services and implementation of safety measures.

#### Consumer perception

Whether consumers support participation in spectator sport consumption comes from attitude theory. That is, consumers’ attitudes toward spectator sport consumption determine whether they support spectator sport consumption. This study, based on interview data, shows that consumers’ perceptions of spectator sport consumption are the main cognitive factors influencing their participation.

Perception of spectator sports consumption is the extent to which the public understands its content and is an significant factor in influencing their attitudes. The theoretical framework of rational conduct underlines the salient role of personal attitudes in shaping behavioral inclinations. Thus, the depth of consumers’ perceptions of spectator sport consumption can be considered have a strong connection to whether they participate or not. Consumers have a short-sighted cognitive bias. When consumers’ attention of spectator sport consumption increases, their participation should also increase accordingly.

Consumers’ perceptions of spectator sports consumption also influence their support for them. In China, spectator sports have a smaller audience than participatory sports and physical sports, so it is difficult for consumers to make purchasing decisions when they only have a vague concept of spectator sports and lack in-depth knowledge about them. The extent of consumers’ cognizance regarding spectator sports consumption serves not only as a pivotal catalyst in spurring purchase intent, but also the crux in reconciling the schism between attitude and behavior. Via qualitative examination, we ascertained that consumers’ cognition of spectator sports consumption mainly includes consumption backgrounds, consumption goals and consumption results.

### Contextual factors

Involvement in spectator sports consumption is readily catalyzed or swayed by the external milieu. Grounded in qualitative examination, we ascertained the contextual parameters impacting consumer participation encompass three dimensions: external motivation, social influence and urban contextual.

#### External motivation

Market incentives, policy incentives and information content are the key contextual factors influencing consumers’ intention towards spectator sports consumption, and all three are external incentives. Supplementary to harnessing government backing and direction, event operators and related sports product vendors, among others, also compete for market share by attracting consumers through various marketing activities.

Our qualitative examination intimates that enhancing consumers’ cognizance regarding spectator sports consumption can be achieved through diverse channels of intelligence, advertising or promotion, either proactively or passively gleaned, thereby bolstering their advocacy for involvement in spectator sports consumption. Our analysis further suggests that by directly or indirectly experiencing spectator sports consumption in advance, it is possible to reshape purchasing behavior and minimize the “psychological distance” between consumers and spectator sports consumption.

The level of participation in spectator sport consumption is closely linked to policy support. These policies can amplify consumers’ cognizance of regulations and readiness to engage in spectator sports consumption. External policy incentives include government subsidies, taxes, and financing^66^. To sum up, our analysis indicates that government should contribute to enhancing consumers’ intention to engage in spectator sports consumption.

#### Social influence

Individual attitudes have been shown to be influenced by social factors, which is an important factor influencing consumer decision-making in China. As the number of high-level sports events held in China continues to increase, consumers’ attitudes toward spectator sports consumption will continue to change under the power of society. The results of this study show that the social factors that influence Chinese consumers’ participation in spectator sports consumption mainly include sports environment, peer pressure and social capital.

The sports environment is the natural and social conditions on which sport exists and develops, as well as the interrelationships between them, and is “the synthesis of all natural and social conditions that are interconnected, mutually constraining and mutually reinforcing” between individuals and sport^67^. Bandura’s triadic determinism views the environment as causally related to people and their behavior, and as having a two-way deterministic and interactive relationship^68^. The results of our interviews revealed that the sports environment that influences the public to engage in spectator sports consumption mainly consists of natural conditions and the number of sports events. Both have a significant impact on the public’s intention to participate in spectator sport consumption.

Peer pressure, as a critical dimension of social influence, plays a particularly vital role in shaping individual support for spectator sports consumption. Meanwhile, innovation diffusion theory intimates that the intrinsic impact factor in contagion prototypes wields greater potency among populations of analogous social standing. Specifically, when consumers observe their peers engaging in spectator sports consumption of a particular sport, they are influenced to adopt the same behavior. Critically, consumer predilections and cravings are swayed by the predilections and cravings of their cohorts^69,70^. When a consumer knows that a friend enjoys a particular sporting event, his or her intention to participate in spectator sports consumption is subconsciously facilitated.

The results of our qualitative analysis suggest that interests, social relationships and social networks are the main social capital factors that influence the public to engage in spectator sport consumption. Profitability is one of the reasons why people engage in an activity, and people tend to gravitate to what is good for them.Research on social networks has shown that social relationships can have an impact on consumers in both the long and short term time frames^71^. Thus, consumers’ social capital is critical to whether they engage in spectator sport consumption.

#### Urban contextual

The vastness of China has resulted in economic and cultural differences in different regions, and has also led to differences in the intention of residents in different regions to participate in spectator sports consumption. Numerous examinations have corroborated that the attributes of the municipality where consumers reside impact their behavioral designs^54^. Predicated on the inferences of the interviews, we found that urban contextual factors such as the level of development of the city, the configuration of sports facilities and the natural environment affect people’s attitudes towards spectator sports consumption.

Since China embarked on reform and opened up, the country has enjoyed rapid development, but the problems of imbalance and inadequacy in the process of development have also been exposed, with the imbalance in the economic development of different regions being particularly prominent^72^. This gap directly contributes to the divergent innovation capabilities and financial expenditures across cities. This divide will also invariably impact consumer behavior on a micro level. Specifically, there exists a pronounced difference in consumers' intention to engage in spectator sports consumption between cities boasting varying development levels.

The configuration of sports facilities affects not only public health, but also the consumers’ intention to participate in spectator sports consumption. Accordingly, the adequacy of the allocation of urban sports facilities, especially venues capable of hosting high-level sports events, affects the public’s perception of spectator sports consumption. Such perceptions can positively or negatively influence consumers’ intention to spend and their consumption behavior. According to our interview data, most of the respondents with negative attitudes toward spectator sports consumption are located in cities with poor or relatively outdated sports configurations.

China is a vast country, and different regions have different geological landscapes, so their sports development process is unique, which also leads to differences in sports consumption in different regions. Specifically, the low temperatures in northeastern China create unique conditions for the development of ice and snow sports events; the development of water sports events in coastal cities such as Qingdao is also highly advantageous, while western China, such as Xinjiang and Tibet, are not suitable for sports events due to the high altitude and thin oxygen.Our interview data show that people living in Northeast China are more likely to go to ice and snow sports events, while people living in West China rarely go to sports events. Hence, we included the geography of the city in our model of consumers’ intention to participate in spectator sport consumption.

### Product factors

The quality of the product has been shown to act as a crucial component of the consuming process. The product of spectator sports consumption refers to the sporting event itself, as well as the quality of the product essentially corresponds to the ornamental value and entertainment value of the event. Therefore, the quality of sports events is closely related to the public’s intention to participate. This study found that event attributes and expenses are the main product factors that influence the public’s intention to participate in spectator sport consumption.

Variations in event attributes serve as a key reason for differences in consumer choices given the same stimulus. The organization of premier sports events or the involvement of star athletes can greatly attract public interest. Concurrently, stadium safety and standardized event organization substantially influence public participation in spectator sports consumption. Scientific and rational event organization can optimize the consumer experience. Consequently, our study shows that high-level sports events and safe event organization can effectively attract the public to spectator sports consumption.

The impact of spending on spectator privileges, such as tickets, and other expenses (clothing, food, etc.) during the course of watching a sporting event on the public’s intention to participate in spectator sports consumption should not be overlooked.Excessive ticket prices for regular, high-level sporting events are a major deterrent to consumers coming to watch them.For example, “ I think tickets are too expensive, so I don’t really want to pay so much to watch a regular game.” Therefore, we included product use spending and other spending in our model of consumers’ intention to participate in spectator sport consumption.

### Analysis of logical relations

**Figure 3:**
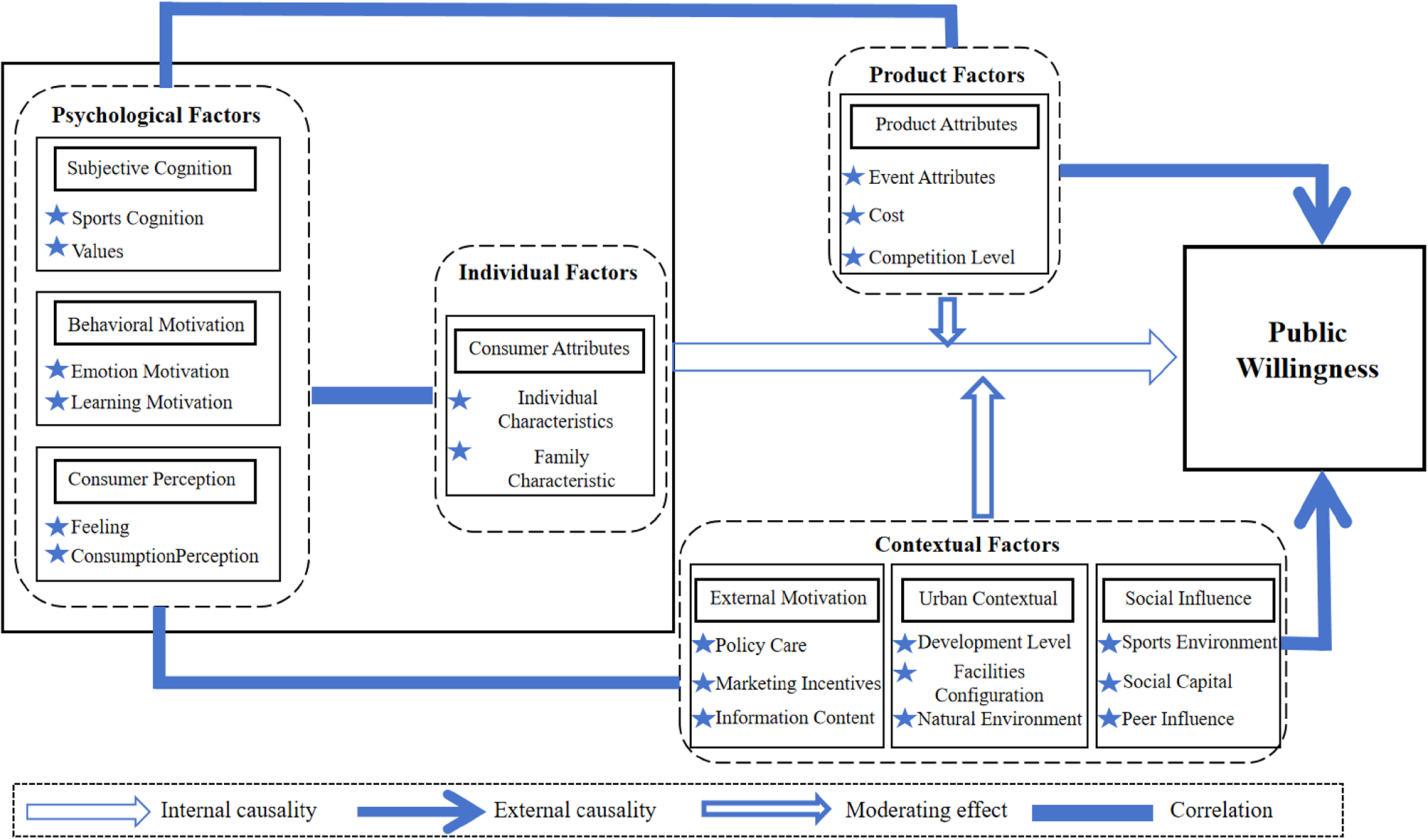
The logical relation model.

Based on the grounded theory and utilizing the original interview data, this study established a model of the public’s intention to participate in spectator sports consumption, which contains a total of four core factors: individual, psychological, contextual and product. The model synthesizes theoretical findings on spectator sports consumption from diverse perspectives, while also elucidating novel categories and relationships. The rational interconnection between these eight constituents is depicted in Fig. 3**.**

Both individual and psychological factors are intrinsically causally related to consumers’ intention to participate in spectator sport consumption, and both can influence consumers’ intention to participate individually or in combination. Taking the consumer attribute variables as an example, it is found that personal characteristics and family characteristics are closely related to whether or not consumers actively participate in spectator sports consumption. Generally speaking, when individuals themselves are very fond of sports, or their families have a strong sports atmosphere, then they are likely to develop a strong willingness to participate in sports consumption. Internal elements, chiefly psychological components, are exceptionally vulnerable to the sway of extrinsic factors in their capacity. Consumer psychological factors are more likely to be influenced by situational and product factors than individual factors. Taking the urban environment variable as an example, it is found that the level of development and the configuration of sports facilities in the city where consumers live are closely connected with their perceptions of spectator sports consumption. Due to China’s national conditions, there are significant differences in the economic development levels of different provinces and cities, and the development of sports in different regions is closely related to the local economic level. As a result, there is a big gap between the sports atmosphere presented by different cities. In general terms, when consumers detect a strong sports atmosphere in the city, they will be interested in spectator sports consumption, which leads to a stronger intention to participate in spectator sports consumption.

Contextual factors can directly affect consumers’ intention to participate, while also indirectly influencing intention by shaping psychological factors. Exogenous catalysts like external motivation and social influence have been identified as external drivers of policy implementation and entry points for changing negative public intentions. Taking external motivation as an example, when policies such as “Strong Sporting Nation” and “Healthy China” are enacted, the public pays more attention to sports in order to respond to the call of the State, which will subconsciously change their understanding of sports and motivate them to actively participate in sports consumption. External factors encompass not just contextual elements, but also critical product factors that help account for differences in consumer intention^54^. When the degree of enjoyment of sporting events meets consumers’ expectations, including perceived entertainment value, atmosphere, skill level, and economic benefit. Taking the attributes of events as an example, with the “explosion” of low-grade events such as the “Village BA” in Taipan Village, Guizhou Province, and the “Village Super” in Rongjiang and Meixiang Villages, Guizhou Province, it can be seen that sports events in line with the local culture and customs are better able to reflect their own characteristics, which makes people feel a sense of belonging, and people are more willing to engage in sports consumption. Similar to previous studies^73^, our research demonstrate that the external factors in the motivational model not only exhibit direct influence, but also display a lightening role when internal factors come into play.

## Research conclusion, limitation and prospect

### Main conclusions

This study is a preliminary study, and we analyzed and coded the text of semi-structured interviews with 25 individuals using a rooted theory research methodology. The cardinal deductions are delineated below.

1. Variables influencing consumers’ propensity for engagement in spectator sports consumption can be classified into individual, psychological, contextual, and product categories. Among these, individual and psychological constituents represent critical innate determinants impacting consumers’ intention, while contextual and product factors constitute external driving factors.
2. Psychological and individual factors are internal factors that influence consumer participation in spectator sport consumption. Psychological factors mainly include subjective cognition, behavioral motivation and consumer perception. Individual factors mainly include individual characteristics and family characteristics.
3. Product and contextual factors are external factors that influence consumer participation in spectator sport consumption, and they moderate internal factors.Product factors mainly include event attributes, cost and competition level. Contextual factors mainly include external motivation, urban contextual and social influence.
4. The dimensions within the four primary factors impacting intention to engage in spectator sports consumption were determined to be independent; they had a combined influence through partial superposition in addition to acting independently on public support.

### Research limitations and outlooks

Spectator sports consumption serves as a vital component for the development of the sports industry. Although this study summarizes the factors that influence the public’s intention to participate in spectator sports consumption and lays a theoretical foundation for how it should develop, there are still shortcomings due to the limitations of the scope of the study.

Specifically, the scale of the survey is limited, and the external validity of the findings needs to be tested by a larger survey. This study has only constructed the theoretical framework of the consumer participation motivation model, it has not been empirically tested.With this theoretical framework, future research should focus on the openness and quantification of measurement scales. As an exploratory study, we only considered consumers’ attitudes towards spectator sport consumption, ignoring the economic benefits and emotional value generated by consumers’ participation. As a result, we are unable to provide clear evidence for the sustainability of spectator sport consumption, which needs to be explored further.

## Data Availability

The datasets used and/or analyzed during the current study available from the corresponding author on reasonable request.
